# Clinical spectrum of Celiac Disease in adults at a tertiary care hospital in Karachi, Pakistan

**DOI:** 10.12669/pjms.38.3.4446

**Published:** 2022

**Authors:** Verda Arshad, Maha Inam, Safia Awan, Faisal Wasim Ismail

**Affiliations:** 1 Dr. Verda Arshad, MBBS. Research Fellow, Department of Medicine, Mayo Clinic, MN, USA; 2 Ms. Maha Inam, MBBS. Medical Student, Medical College, Aga Khan University, Karachi, Pakistan; 3 Ms. Safia Awan, MSc Statistics, Senior Instructor, Department of Medicine, Aga Khan University, Karachi, Pakistan; 4 Dr. Faisal Wasim Ismail, FCPS Gastroenterology, Associate Professor, Department of Medicine, Aga Khan University, Karachi, Pakistan

**Keywords:** Autoimmune Diseases, Celiac Disease, Diet, Gluten-Free

## Abstract

**Objectives:**

Celiac Disease (CD) is a disorder that impacts physical, social and emotional health. Requiring life-long treatment, it poses a major economic burden on the healthcare system. Our objective was to study CD in patients from initial presentation to diagnosis and to ascertain the effect of a low resource setting on improvement in disease process.

**Methods:**

This is a retrospective cross-sectional study conducted at a Aga Khan University Hospital (AKUH), a tertiary care center in Karachi, Pakistan. Medical records of patients (≥ 18 years) from 2008 to 2018 with a diagnosis of CD were reviewed. Data on demographics, presenting complaints, investigations, endoscopy results and follow up visits was collected.

**Results:**

One hundred and twenty-six patients were included (61.6% females, mean age 35.5 years). The most common intestinal and extra-intestinal symptoms were abdominal pain (56.3%) and fatigue (24.6%) respectively. After microcytic anemia (36.5%), increased ALT (27.2%) was the most common laboratory derangement. On endoscopy, visible fissuring (29.4%) and atrophic mucosa (29.4%) were reported. Biopsy findings showed increased intraepithelial lymphocytes (92.9%) and villous atrophy (77.8%). Improvement in at least one of three parameters (symptoms, laboratory values or EGD) was reported by 42.0% of subjects, whereas 48.4% subjects were lost to follow-up.

**Conclusion:**

The most commonly reported symptoms by CD patients were abdominal pain, diarrhea and anemia. Thus, patients presenting with vague abdominal symptoms and anemia should be worked up for CD. A concerning majority of subjects was lost to follow up for reasons such as inability to afford advised GFD and a poor understanding of the disease process.

## INTRODUCTION

Celiac Disease (CD) is an immune-mediated systemic disorder that primarily affects the gastrointestinal tract but has widespread manifestations affecting the integumentary system, central nervous system, reproductive system and musculoskeletal system. Development of the disease occurs due to exposure to diet containing gluten in genetically predisposed individuals. CD is diagnosed based on presence of disease specific antibodies in the serum and small intestinal mucosal biopsies. Avoidance of gluten in diet is the only known treatment of this disease. Untreated CD in the long run increases the risk of benign and malignant conditions, and mortality.^1^

Globally, the prevalence of CD based on serologic test results is 1.4% and based on biopsy results is 0.7%.^2^ CD is a poorly studied disease in most Asian countries because until a few decades ago it was considered to be a disease that affected individuals with European ancestry. Based on the scarce data that is present, the overall prevalence of CD in Asian countries is 0.5%.^3^ While these numbers may not look alarming, there are multiple reasons to suggest that the actual prevalence of the disease is much higher. It has been shown that almost 50% of newly diagnosed CD patients have an asymptomatic clinical course^4^; based on this, it can be reasonably extrapolated that countries without screening programs have a higher disease burden than reported. Furthermore, recent studies have reported an increasing trend when studying the temporal changes in frequency of CD.^5^

CD has drawn a lot of attention in recent years. As a disease that requires life-long treatment and follow up, it poses a major economic burden on the healthcare system of any country. Studies conducted have estimated cost per positive CD diagnosis ranging from $1,300 in Canada to more than € 44,000 in the Netherlands.^6^ Additionally, it is a disease that significantly decreases the quality of life of those affected by decreasing functionality in social relations, emotional life, physical and psychological health.^7^

Currently, there is a lack of comprehensive literature on CD in Pakistan covering epidemiology, clinical presentation and treatment. A systematic review reported that there were only 14 clinical studies on CD in adults in Pakistan of which seven were case reports.^8^ To work on this gap that has been identified, the aim of this study was to look at patients presenting with CD, in all its entirety, starting from initial presentation to diagnosis and then follow-up. The authors also aim to ascertain compliance and improvement in patients in a low resource setting.

## METHODS

This is a retrospective observational study conducted in Aga Khan University Hospital (AKUH), a tertiary care center in the most populated city of Pakistan, Karachi. Ethical exemption was granted by the Ethical Review Committee of our institution on 27^th^ December, 2018 (approval number: 2018-0785-1066).

Medical records of patients above the age of 18 with a diagnosis of CD at AKUH between the years 2008 and 2018 were reviewed. For each medical record, a questionnaire was filled out, which included the following data: demographics; intestinal and extra intestinal signs and symptoms of CD; laboratory parameters before the diagnosis of CD; antibody titers and esophagogastroduodenoscopy (EGD) and biopsy findings. To study compliance and response to a gluten free diet (GFD), the questionnaire also included post diagnosis improvement in symptoms experienced by the patients and changes in laboratory parameters, antibody titers and EGD results on follow-up. The questionnaire was tested on the first 10 medical records and after modifications, it was finalized. (See Appendix: Celiac Disease Questionnaire)

Diagnosis was based on the American College of Gastroenterology clinical guidelines. CD was detected by serologic testing of celiac-specific antibodies and diagnosis was confirmed by duodenal mucosal biopsies.^9^ To ascertain a positive response to gluten free diet (GFD) we included three aspects of every patient’s follow-up in our questionnaire i.e. symptomatic improvement, improvement in laboratory parameters, and improvement on repeat EGD and biopsy. If there was an improvement in any one of these aspects, the patient was labeled as showing a positive response to GFD.

### The exclusion criteria were

(1) diagnosed cases of CD, or those already on a GFD (2) records with incomplete data (3) patients who were lost to follow up before a definitive diagnosis was made. All other medical records were included in the study. Data was analyzed using SPSS v.19.

## RESULTS

This study included a total of 126 patients diagnosed with Celiac Disease between 2008 and 2018 at AKUH. Of these 38.9% (n=49) were male and 61.1% (n=77) were female. The mean age was 35.5 ± 12.3 years.

 Concomitant autoimmune conditions were found in 19.0% of the subjects; among which hypothyroid (41.7%) was the most common, followed by psoriasis (25.0%), Type-1 diabetes mellitus (16.7%), autoimmune hepatitis (12.5%), systemic lupus erythematosus (8.3%), and others including hyperthyroid, idiopathic thrombocytopenic purpura and polyarteritis nodosa.

**Fig.1:**
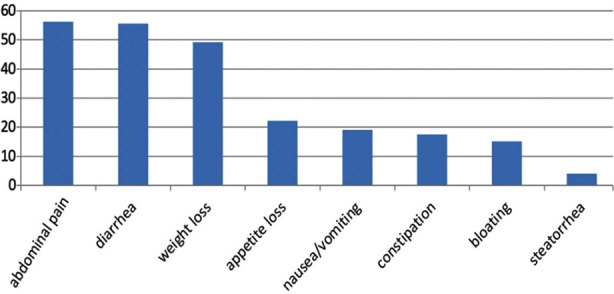
Intestinal manifestations reported.

**Fig.2:**
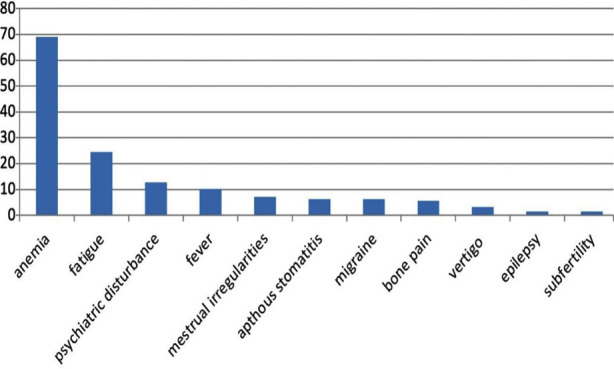
Extra-intestinal manifestations reported.

Symptoms experienced by patients were divided into intestinal (Fig.1) and extra-intestinal symptoms (Fig.2). The most common intestinal symptom reported was abdominal pain (56.3%); others included diarrhea (55.6%), unexplained weight loss (49.2%) and decreased appetite (22.2%). Common extra-intestinal signs and symptoms experienced were anemia (69.0%), fatigue (24.6%) and fever (10.3%). Psychiatric disturbances were reported in 12.7% of the subjects with depression (37.5%) and anxiety (25.0%) as the most commonly reported conditions. Multiple obstetric and gynecological problems were also reported including menstrual irregularities (11.7%), sub fertility (2.8%), recurrent fetal loss (1.4%) and low birth weight neonates (1.4%).

Overall, anemia was the most common lab abnormality. Mean hemoglobin in females was 10.2, whereas in males it was 11.97. Microcytic anemia was the most common followed by normocytic anemia (Table-I). Menstrual irregularities were excluded before attributing the anemia to CD. Alanine transaminase (ALT) levels were found to be elevated in 27.2%. In females, the average ALT value was 34.7 IU/L and in males it was 36.5 IU/L. 10.9% of the subjects reported low Vitamin B12 values.

Anti-tissue transglutaminase (anti-ttg) IgG level and IgA serological testing showed a mean value of 33.9 (SD=50.0) and 106.1 (SD=123.1) respectively. Of those who got serological testing for anti ttg IgA done (n=108), IgA levels were negative in 12.1% (n=13), intermediate in 0.9% (n=1) and positive in 87.0% (n=94). Similarly, those who tested for anti ttg IgG levels (n=105), levels were negative in 18.1% (n=19), intermediate in 0% and positive in 81.9% (n=86). Overall, of those who got serologies done, 8.3% (n=9) were seronegative.

All subjects, except one, underwent esophagogastroduodenoscopy (EGD). Gross findings on EGD were visible fissuring and scalloping (29.4%), atrophic mucosa (29.4%) and nodularity (4.0%). Biopsy results showed increased intraepithelial lymphocytes (IEL) (92.9%), villous atrophy (77.8%) and crypt hyperplasia (5.5%). Helicobacter pylori gastritis was seen in 73.9% (n=82). HLA typing was done in only one subject, who was found to be HLA-DQ2 positive.

**Fig.3:**
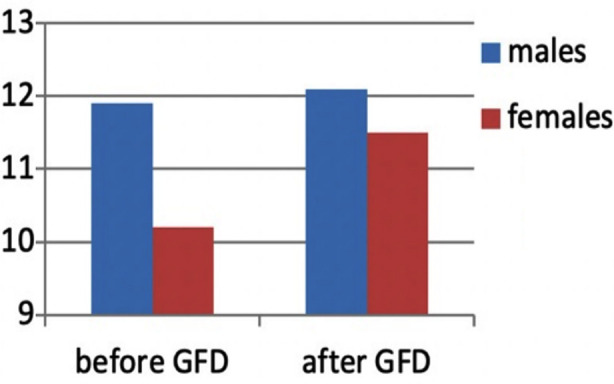
Comparison of mean hemoglobin levels (g/dL) before and after starting GFD.

When studying the outcome, 48.4% of the subjects were lost to follow-up, 42.0% reported a positive response to GFD, 4.8% were non-compliant and another 4.8% failed to show a positive response after commencing GFD. The authors assessed improvement based on three criteria: improvement in symptoms, improvement in laboratory values or improvement upon repeat EGD. Symptomatic improvement was reported by 21.4% subjects. Improvement in laboratory values was seen in 34.1%. This included anti-ttgIgG level improvement in 20.6%, anti-ttg IgA level improvement in 24.6%, hemoglobin level improvement in 19.8%. Fig.3. ALT level improvement in 1.6%. Lastly, improvement upon repeat EGD was seen in 4.8% of the subjects.

## DISCUSSION

Celiac Disease affects not only the physical health of an individual but also their mental wellbeing and social life. This makes living with the disease very difficult for those suffering from it, significantly impacting the quality of life. With limited data on the disease in Pakistan, we need to increase our efforts to understand the disease better in the context of our population.

The mean age of subjects was 35.5 ± 12.3 years. This is comparable to previous studies conducted in Pakistan showing mean ages of 29.9 ± 12.7 years^10^ and 38±10.5 years.^11^ These statistics signify that the age at diagnosis in Pakistan varies between the late 20s and 30s.This is similar to the age of presentation in Iran in the middle-East and Italy in Europe but strikingly different from the USA, where age of diagnosis is between fourth to sixth decades of life.^12^,^13^ In this study it is reported that the disease affected females more than males with a female to male ratio was 1.6:1, much lower than an earlier study conducted in Hyderabad, Pakistan showing a ratio of 3:1.^11^

Celiac Disease was once thought to be a disease of the gastrointestinal tract. As knowledge on the disease has expanded the medical community now knows that it can affect almost any organ system of the body. To make diagnosis even more difficult, sometimes there are no symptoms reported. These factors make celiac disease a particularly tricky disease to diagnose. Anemia was the most commonly reported extra-intestinal manifestation in this study. With 69% subjects showing anemia, it is overall the most common manifestation of CD in the subjects. Other studies have shown even higher levels with 83.3% patients reporting anemia.^14^ Additionally, a study conducted in the same center in the past has shown that in patients presenting with iron deficiency anemia, an alarming 11% were diagnosed with CD.^15^ Given all this data, it is important for physicians to keep CD on the list of differentials in a patient presenting with anemia.

The most common intestinal symptom reported was abdominal pain. This is in contradiction with earlier studies that reported diarrhea, affecting 50 to 69.4%,^10^,^11^ as the most common intestinal symptom in Pakistan. Diarrhea was present in 55.6% of our subjects, falling within the range reported by earlier studies. A myriad of other intestinal symptoms was reported including nausea and vomiting, constipation, bloating, unexplained weight loss, steatorrhea and decreased appetite. Often times unexplained gastrointestinal symptoms are diagnosed as Irritable Bowel Syndrome (IBS). Indeed, a study conducted among physicians in Pakistan to investigate their knowledge of the differences between IBS and CD that CD is often misdiagnosed as IBS.^16^ Thus, in Pakistan it is pertinent to investigate unexplained and vague gastrointestinal symptoms in adults by conducting serologic tests for CD.

Previous studies conducted to study EGD findings in Pakistan have been small scale studies. The most common EGD findings in this study were visible fissuring /scalloping and atrophic mucosa whereas the most common biopsy finding was increased IELs. However, a previous study conducted on 39 patients showed villous atrophy as the most common biopsy finding.^10^ Once again this shows that CD in its entirety can present in a multitude of different ways. However, given how most previous studies have been conducted on a small scale and this study encompasses only one healthcare center, we need more large-scale studies to improve understanding.

To treat and control CD, a patient has to take GFD all their life with regular follow-ups and repeat testing. The significance of GFD in improving the quality of life can be gauged by a study that showed that even CD patients with no initial symptoms reported an improvement in symptoms after taking GFD.^4^ However in resource limited settings, this becomes increasingly difficult for patients making compliance and regular follow ups a problem. In Pakistan, gluten free (GF) products are mostly imported and thus expensive. Additionally, regulations regarding accurate food listing are not observed allowing contamination of products labeled as GF.^17^ This could explain why in this study more than half the patients were either lost to follow up or non-compliant. Raising awareness will help increase investment in locally produced GFD which can be cheaper and more easily accessible. The effect of raising awareness is two-fold as it will also allow those with the disease to understand the treatment better, making it easier to follow. Additionally, large-scale studies should be conducted on the understanding of patients on GFD and the challenges they face in acquiring it.

Pakistan is a low-middle income country. Investment in healthcare is low and very few people have medical insurances to bear the burden of their healthcare. The effect of limitation in resources can be seen in various different aspects of our study. Apart from the low compliance discussed in detail earlier, testing on initial visits and follow-up is curtailed according to the affordability of a patient. The effect of that is that only one patient has been tested for HLA genes. Only 10% of the subjects underwent repeat endoscopy even though it has been seen that ensuring complete mucosal healing decreases the chance of malignancy in the future. Thus, on a bigger scale, studying the impact of diseases like CD and raising awareness about them can encourage increased investment in healthcare. This can help decrease the financial burden on patients suffering from conditions like CD, assisting in their management.

## CONCLUSION

Upon presentation, patients with vague abdominal symptoms and anemia should be worked up for CD. A concerning majority (48.4%) of subjects was lost to follow up for a variety of reasons including inability to afford advised GFD and a poor understanding of the disease process. More studies need to be conducted on a larger, population-based scale to improve our knowledge on the spectrum of CD and compliance to GFD in Pakistan.

### Authors Contribution:

**VA:** Conducted the literature search, data collection, manuscript write-up and proof reading.

**SA:** Did the data analysis and data interpretation.

**MI:** Contributed towards the manuscript write-up, editing and proof reading.

**FWI:** Conceptualized the study design and conducted the manuscript write-up and proof reading. Responsible and accountable for the accuracy and integrity of the work.
